# Dynamics and development of interhemispheric conflict solving in pigeons

**DOI:** 10.1038/s41598-024-85058-9

**Published:** 2025-01-11

**Authors:** Martina Manns, Kevin Haselhuhn, Nadja Freund

**Affiliations:** 1 Division of Experimental and Molecular Psychiatry, Department of Psychiatry, Psychotherapy and Preventive Medicine, LWL University Hospital, Ruhr- University, Bochum, Germany; 2 Department of Biopsychology, Faculty of Psychology, Institute of Cognitive Neuroscience, Ruhr-University Bochum, Bochum, Germany

**Keywords:** Bird, Avian, Lateralization, Metacontrol, Ontogenetic plasticity, Decision-making, Cognitive neuroscience, Visual system

## Abstract

The dominance of one hemisphere for cognitive operations and decision making may be an efficient mechanism solving interhemispheric conflicts. To understand the ecological significance of the so-called metacontrol, we need better knowledge of its frequency and ontogenetic foundations. Since in pigeons, embryonic light experiences influence degree and direction of interhemispheric specialization and communication, it is conceivable that light affects metacontrol mechanisms. We therefore trained pigeons (*Columba livia*) with and without embryonic light stimulation in a colour discrimination task. Each eye/hemisphere learnt a different set of colours. After training, hemispheric-specific information was put into conflict and the analysis of conflict decision pattern allowed the identification of hemispheric dominance under binocular and monocular viewing conditions. A majority of pigeons displayed individual metacontrol independent of embryonic light experiences though not in the first test session. Reaction times indicate that interhemispheric mechanisms are critically involved in mediating the dominance of one hemisphere. The impact of interhemispheric components rises with increasing experience and even affects decision making under monocular seeing conditions. Overall results indicate that the hemispheres do not evaluate information independently and that interhemispheric communication in the pigeon brain is much stronger than previously thought and becomes more important with increasing experience.

## Introduction

The nervous system of bilateral animals typically consists of two halves, each primarily processing sensory input from one half of the environment and controlling one side of the body^1^. When due to the organization of the sensory systems, transmission of bilateral sensory information is limited, the left and right brain side receive different inputs and the brain must determine their respective relevance to organize attention focus and to decide on an appropriate behavioural response.

In many animal species, the two hemispheres of the brain are specialized to take over different functions, which increases the available computational capacities and enables coping with two tasks simultaneously. Such functional asymmetries are based on structural and physiological left–right differences in neuronal networks and lead to a complex pattern of lateralization with differential hemispheric dominance for certain aspects of perception, cognition and behaviour. This means that the hemispheres consider different aspects of the environment, attend to different sensory cues, and differ in information processing style and decision-making^2–5^. In vertebrates, the left hemisphere prefers a serial processing style relying on local or high-frequency aspects of stimuli, while the right hemisphere favours parallel or configural processing, encoding global or low-frequency information. These preferences are related to hemispheric functional specialization whereby the left hemisphere typically dominates routine behaviour including foraging, discrimination of food objects, or prey catching, while the right hemisphere controls emergency responses and is therefore in charge of novelty or predator detection as well as in visuospatial attention^2,4–6^. However, a lateralized brain organization may cause interhemispheric conflicts when objects of equipotential importance appear in the left and right hemispace or when the two hemispheres process and evaluate a situation differently so that they evoke incompatible response tendencies. Since such conflicts slow down decision-making and behavioural responding and hence, are of potentially evolutionary disadvantage, there should be high selection pressure on the presence of neuronal mechanisms solving interhemispheric conflicts.

Interhemispheric conflicts are particularly likely in birds with laterally positioned eyes like chicks or pigeons, which have only a narrow frontal binocular field^7^. Here, owing to the almost complete crossing of the optic nerves, the right eye mediates visual input to the left and the left eye to the right hemisphere^8^. Commissural fibres within the visual pathways^9–11^, allow some bilateral transfer of visual input, hemispheric exchange of visual information^12,13^ and integration to solve cognitive problems^14,15^. However, birds preferentially eye objects in the environment with their lateral monocular visual fields before eventually approaching them^16^. This implies that the avian brain needs neuronal mechanisms, which control interhemispheric attention switch and eventual suppression of input from one eye. To this end, inhibitory mechanisms regulate transfer or suppression of interocular information already at lower level of the processing stream^2,17^. On the other hand, interhemispheric mechanisms regulating cognitive operations and decision-making could play a role at a higher level of processing. Originally human research demonstrates the existence of a choice mechanism, called metacontrol that determines which hemisphere controls a task when the two sides of the brain are facing incompatible behavioural options^18,19^. While metacontrol is well described in humans^20^, there are only a few studies conducted in a limited number of animal species like monkeys^21^, chicken^22^ and especially pigeons^23–27^. In pigeons, individual birds display metacontrol when confronted with conflict decisions based on colour discrimination^23,24,26^ or categorization^25^. Results are, however, inconsistent concerning degree and direction of hemispheric dominance within the tested pigeon groups. This indicates that metacontrol is possibly not fixed and can be affected by factors like task conditions or the situational context, as well as developmental or learning experiences^20^.

When we want to understand the evolutionary relevance of metacontrol, we have to explore potentially modulating factors and their influence onto the neural mechanisms which underly metacontrol. Recent research indicates that intra-as well as interhemispheric mechanisms can regulate hemispheric dominance for conflict choices^24,25,28–30^. In addition to electrophysiological recordings, the analysis of reaction times can differentiate between these mechanisms^25,26,28,30^. On one hand, metacontrol can result from different processing times of neuronal operations in the left and right hemisphere and is therefore derived from intrahemispheric processes. The hemisphere, which processes sensory input faster, also gains faster control over premotor networks and thus, controls the behavioural response. Since this hemisphere controls motor output in any condition, reaction times for conflict decisions do not differ from that for unambiguous choices. On the other hand, one hemisphere can achieve dominance over conflict decisions when it actively intervenes in the neuronal processing of the contralateral brain side via commissural mechanisms. Because of the additional processing step, interhemispheric components increase reaction times^25,26^. First data show that the relative involvement of intra- and interhemispheric mechanisms is dependent on the current task complexity^25^. In addition, it is conceivable that the seeing conditions have an influence on hemispheric dominance. As indicated above, metacontrol mechanisms should particularly come into play when both hemispheres receive information in parallel and thus, directly compete for decision-making and response control. When seeing with only one eye, the hemisphere contralateral to the seeing eye has direct access to the presented stimuli and therefore should have a processing advantage that leads to its control over the behavioural response.

Apart from the situational context, ontogenetic experiences might affect metacontrol. For chicks and pigeons, it is well known that embryonic light stimulation shapes the lateralization pattern of the visual system and how the two brain hemispheres interact^10,11,31–34^. Light deprivation during embryonic development not only avoids the development of functional asymmetries but also impairs efficient interhemispheric task sharing^35^ and cooperation^14,15^ and prevents the development of asymmetrical projections within the visual pathways so that both hemispheres receive symmetrical visual input from the left- and right-eye system^9,11,32,34^. Therefore, the emergence of interhemispheric metacontrol mechanisms might also depend on the embryonic light conditions.

**Fig. 1:**
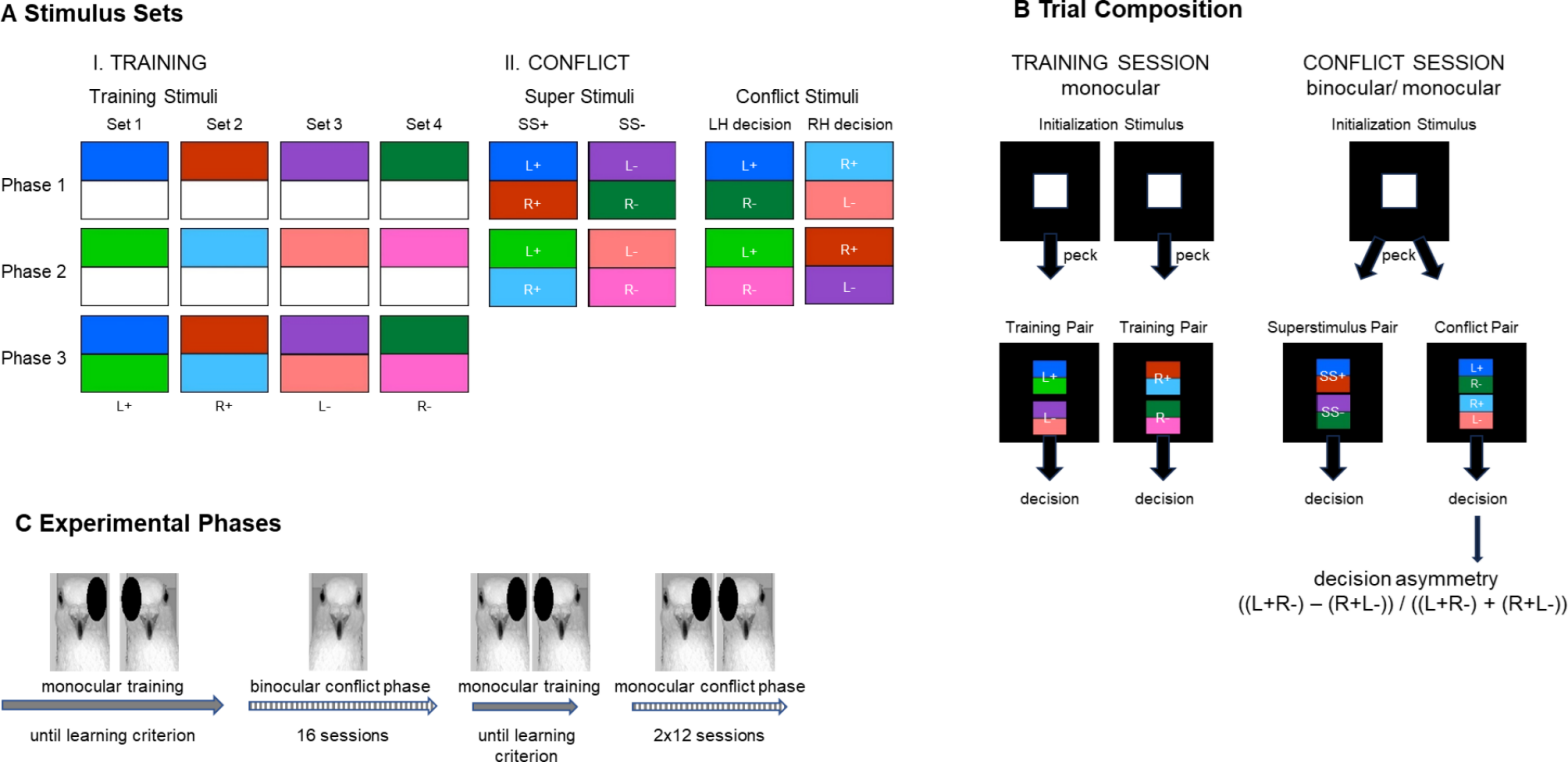
Stimuli and experimental phases—**A** during training phase, four stimulus sets were used as training stimuli and were consecutively introduced during three training phases (Phase 1-Phase 3); during conflict phase, different super and conflict stimuli were presented: positive super stimuli (SS +) combined a colour positive for the left (L +) and right (R +) hemisphere, negative super stimuli (SS −) combined a colour negative for the left (L−) and right (R−) hemisphere (a complete set included four different SS + and SS −). Conflict stimuli combined a colour positive for the left (L +) or right (R +) and a colour negative for the left (L-) or right (R) hemisphere; a complete set included four stimuli correct for the left and four correct for the right hemisphere. Each stimulus was presented in two up-down orientations within a session. **B** Trial composition: each trial of a session started with the presentation of a white square as an initialization stimulus, which had to be pecked once before a colour stimulus pair appeared. During monocular training, the pigeons had to discriminate between stimuli positive ( +) or negative ( − ) for the right eye/left hemisphere (L) or left eye/right hemisphere (R), respectively. Conflict test sessions include trials in which either super stimulus or conflict stimulus pairs were presented. A single peck on one of the conflict stimuli indicated which hemisphere controlled the pecking response (= the hemisphere that had learned the positive colour of the responded stimulus). The percentage of pecks onto the stimulus correct for the left hemisphere (L + R − ) and for the right hemisphere (R + L − ) during a session was used to compute individual decision asymmetry. **C** Test sequence: after the training phase, conflict tests phase started with 16 binocular sessions followed by 24 monocular sessions; blocks of six conflict sessions were always followed by monocular training sessions until a pigeon reached the learning criterion.

To better understand the functional architecture of metacontrol and its ontogenetic origins, we explored the metacontrol pattern of pigeons with and without embryonic light exposure^9,13,36,37^. To this end, we adopted a conflict task, which is based on colour discrimination^24,26,27^. Pigeons were trained to differentiate between positive (rewarded) and negative (non-rewarded) colours with each eye learning different pairs of colours (Fig. 1). Because of the virtually completely crossed retinal projections, each eye is mostly connected to the contralateral hemisphere^8^. We therefore assume that information from the right eye is primarily processed in the left hemisphere (right eye system) and information from the left eye in the right hemisphere (left eye system)^38^, although there are several commissures in the pigeon brain that allow interocular information exchange. As a first approximation, this means that a hemisphere contralateral to an eye is significantly involved in decision-making about the relevance and further processing of information from the contralateral eye.

After monocular training, the pigeons were confronted with pairs of ambiguous stimuli, each combining a positive colour for one eye with a negative colour for the other eye (Fig. 1). Consequently, each hemisphere should decide for a different stimulus, namely the one including the colour with appetitive value, while refusing the one including the negative colour. Pecking at one of the stimuli indicates which hemisphere controls the pecking response, i.e. the hemisphere that learned the positive colour of the responded stimulus. Since embryonic light stimulation affects direction and degree of interhemispheric communication^13,15^, we expected more pronounced metacontrol in light stimulated birds. Apart from binocular tests, we confronted the pigeons with conflict choices when seeing with only the left or right eye to prove whether metacontrol is specific for conflict situations when both hemispheres compete for decision control. We expected individual hemispheric dominance for binocular conflict choices but a decision bias towards stimuli positive for the seeing eye under monocular conditions. This advantage might be more pronounced with the right eye since the left hemisphere is in mean superior and faster in visual processing^28–30,38^.

## Results

### Learning phase

During colour discrimination training, pigeons learned to discriminate the positive and negative colour stimuli under monocular seeing conditions (Fig. 1A, B). Training was divided into three phases during which the different colour stimuli were consecutively introduced (see Methods section). On average, pigeons required 67 +/− 27 sessions until reaching the learning criterion of more than 85% correct responses with both eyes in the third training phase. The number of required sessions did not differ between light-exposed (63 +/− 26) and light-deprived pigeons (71 +/– 30). There was also no difference between the seeing conditions neither in the light-exposed nor in the light-deprived group. Mean discrimination performance when reaching the learning criterion was 92.29 +/− 3.2% correct responses, and the pigeons needed 1.4 ± 0.29 s for a correct response, with no difference between the seeing conditions or experimental groups.

### Conflict phase

After finishing the learning phase, the conflict test phase began (Fig. 1C). During conflict tests, the pigeons were confronted with two types of stimulus combinations—the super stimuli and the conflict stimuli when seeing with both or with only one eye (Fig. 1A, B). For the analysis of decision patterns, 16 sessions under binocular and 12 under each of the two monocular seeing conditions were considered. The pigeons performed more than this number of sessions in each seeing condition (see Supplementary Material 1), but we only considered sessions with an initialisation rate of at least 75%, i.e. the pigeons had pecked on the initialisation stimulus (Fig. 1B) in at least 75% of the trials in a session. This ensured that the pigeons had made a sufficient number of decisions during an analysed session. On average, the pigeons conducted 882 +/– 250 binocular and 594 +/– 192 monocular conflict decisions. In the following, we first describe the super stimuli discrimination, second the conflict decision pattern, and compare bi- and monocular seeing conditions. Since the pigeons showed as many pecking decisions as were analysed in total in previous studies^23,24,26,27^ already during the first session (on average 64 ± 14 binocular and 50 ± 20 monocular decisions), we additionally analysed the first binocular session separately. It is possible that in the first session within which the birds were confronted the first time with the ambiguous stimuli, response pattern differed from the other sessions^26^. A detailed analysis indicates significant changes in response pattern and reaction time over time (Supplementary material).

#### Super stimuli discrimination

**Fig. 2:**
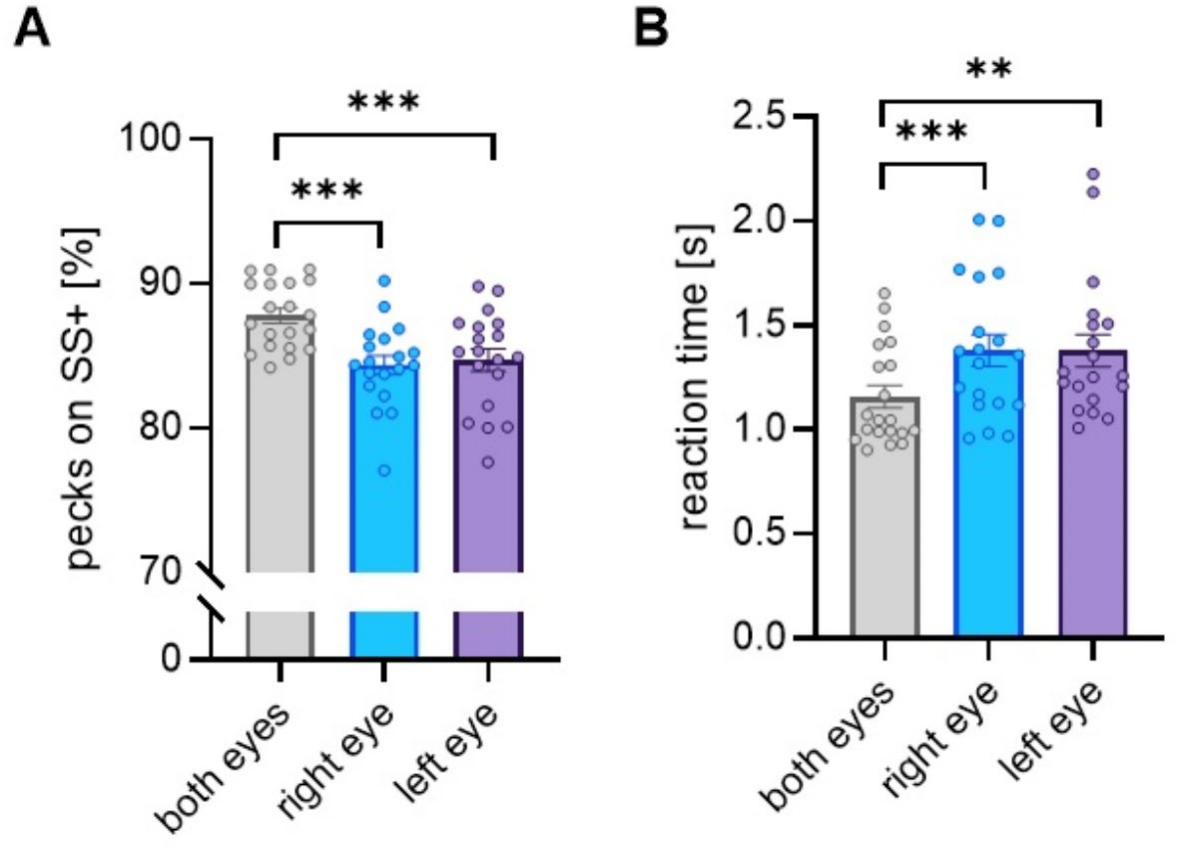
Super stimuli discrimination under different seeing conditions—**A** mean percent decisions for positive superstimuli (SS +); **B** mean reaction time in seconds [s] when pecking onto SS +. Bars indicate standard error, circles indicate individual data; *** *p* < 0.001, **p < 0.01 according to t-tests for dependent samples (**A**) or Wilcoxon tests (**B**).

In 60% of trials within one session, the pigeons had to decide between super stimuli combining either two positive (SS +) or two negative (SS −) colours (Fig. 1A, B). Light-exposed and light-deprived pigeons did not differ in super stimuli discrimination. When seeing with both eyes, the pigeons decided clearly for the SS + (M = 87,89 +/− 2,3%) compared to SS− (M = 2,75 +/− 2,4%; 9,5% +/− 2,4% of the trials were not responded). This was also the case under monocular seeing conditions (SS + : M = 84.54 +/− 3.11; SS− : M = 3.51 +/− 2.16; 12.1% +/− 3% were not responded; Fig. 2A). A repeated measure ANOVA revealed that the percentage of correct responses differed between the seeing conditions (F_2, 16_ = 12,265,44, *p* < 0.000; partial eta-squared η_p_^2^ = 1). While right- and left-eye performances did not differ (t-tests for dependent samples: t = − 0.344, *p* = 0.735), super stimuli discrimination was significantly better under binocular compared to monocular testing conditions (t-tests for dependent samples: bino vs right-eye seeing t = − 4,835, *p* < 0.001; bino vs left-eye seeing: t = − 3.898, *p* = 0.001; Fig. 2A). Response latencies also differed significantly between the seeing conditions (Friedman ANOVA: Chi^2^ (n = 19, df = 2) = 9.579 *p* = 0.008). Binocular response latencies were significantly shorter than monocular ones (Wilcoxon: bino vs right-eye seeing z = − 3.340, *p* < 0.001; bino vs left-eye seeing: Z = − 2.958; *p* = 0.003) but reaction times did not differ when seeing with the left and right eye (Wilcoxon: z = − 0.282, *p* = 0.778; Fig. 2B).

#### Conflict choices

##### Decision patter

**Fig. 3:**
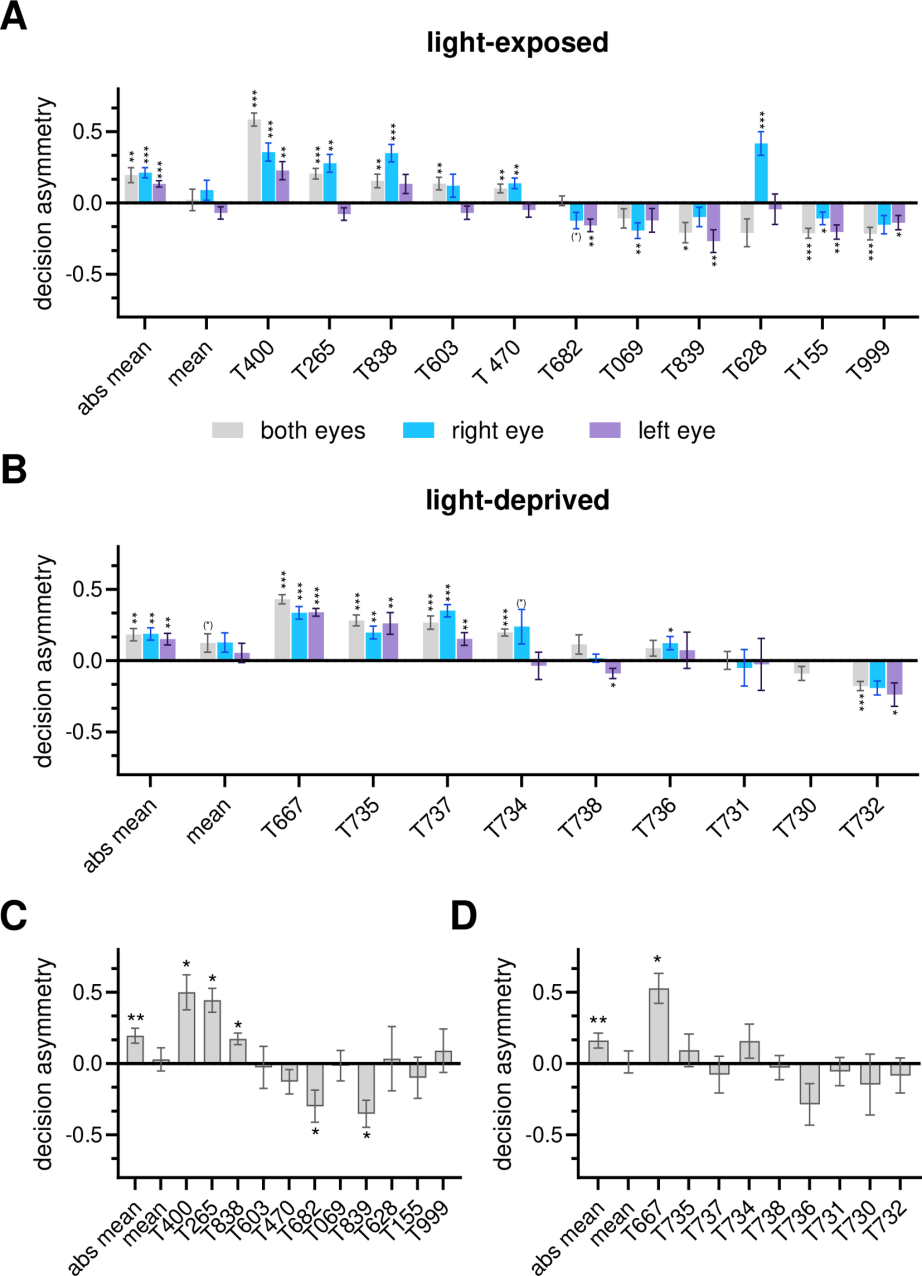
Mean decision asymmetry of all sessions (**A**, **B**) or only the first session (**C**, **D**) in light- exposed (**A**, **C**) and light-deprived pigeons (**B**, **D**) under the different seeing conditions. Depicted are individual values as well as the mean and absolute mean of the experimental groups; positive values indicate left- and negative values right-hemispheric dominance. Bars represent standard error ((*) < 0.1, * < 0.05; **, *p* 0 < 0.01, *** = *p* < 0.001 according to t-test for dependent samples (**A**, **B**) or Wilcoxon tests (**C**, **D**).

**Fig. 4:**
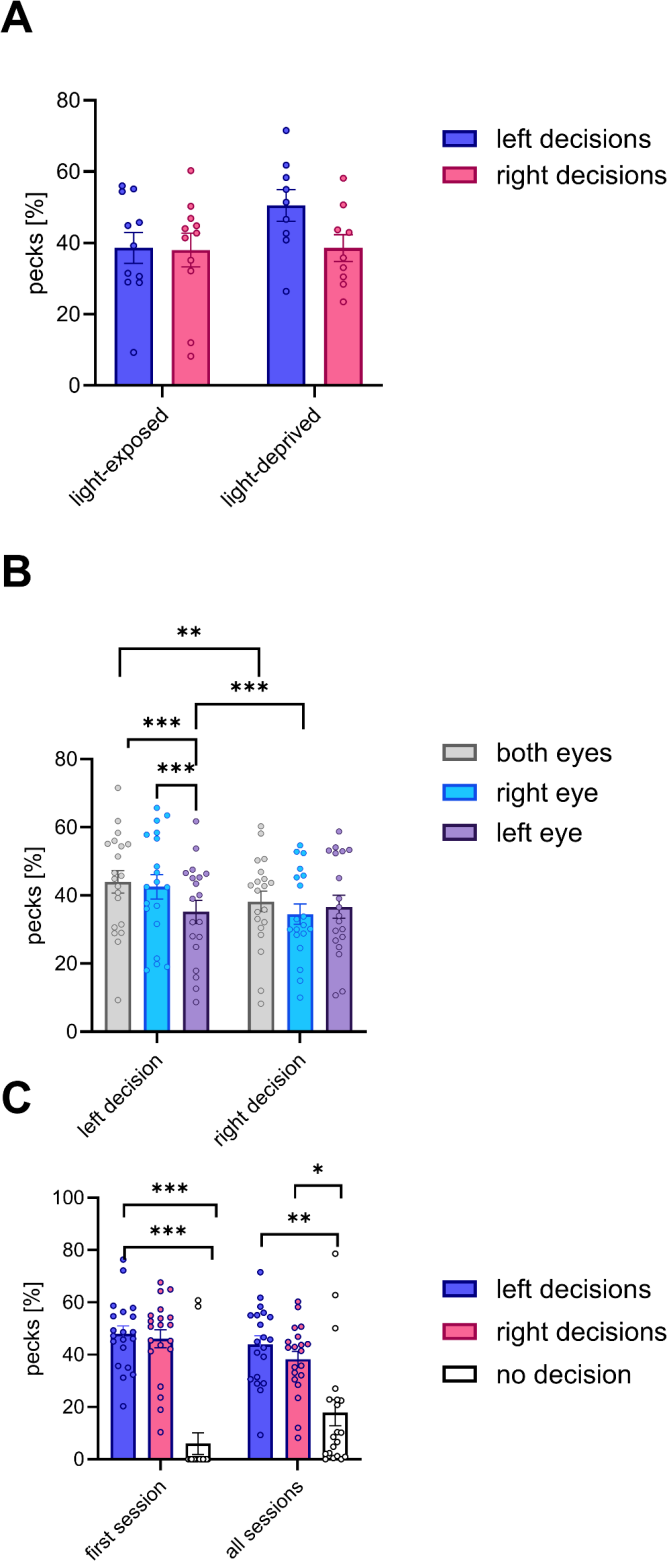
Hemispheric-specific decisions under binocular seeing conditions in light-exposed and light-deprived pigeons (**A**), under different seeing conditions (**B**), during first and all session (**C**). Bars represent standard error; (*) < 0.1, * < 0.05; **p 0 < 0.01, *** = *p* < 0.001 according to posthoc Bonferroni tests (**B**), Wilcoxon test (**C**).

In the conflict trials, the pigeons had to choose between stimuli that were positive for either the left or the right hemisphere. The percentage of pecks onto left- and right-hemispheric stimuli during a session served as the basis for calculating the decision asymmetry and thus, as a marker for hemispheric dominance (Fig. 1B) Under binocular viewing, thirteen pigeons (65%) showed dominance of one of the hemispheres, as indicated by a decision asymmetry which differed significantly from zero, with nine pigeons showing left-hemispheric dominance (positive decision asymmetry) and four showing right hemispheric dominance (negative decision asymmetry; Fig. 3A, B). Accordingly, the absolute value of the mean decision asymmetry differed significantly from zero for both groups (one sample t-test: t = 4.484, *p* < 0.001 (light-exposed group); t = 4.246, *p* = 0.003 (light-deprived group)). Since there was an equal number of pigeons showing a left- or a right- hemispheric dominance in the light-exposed group, there was no significant asymmetry of hemispheric dominance at group level (one sample t-test: t = 0297, *p* = 0.773; Fig. 3A). The higher number of pigeons showing a left-hemispheric dominance in the light-deprived group (Fig. 3B), resulted in a trend for left-hemispheric dominance at group level (one sample t-test: t = 1.935, *p* = 0.089). Although the number of left-hemispheric decisions was higher in light-deprived pigeons (Fig. 4A), analysing the percentage of left-and right-hemispheric decisions depending on the experimental group in a mixed 2 × 2 ANOVA showed no significant interaction between both factors (F_1, 18_ = 1.60, *p* = 0.223; partial eta-squared η_p_^2^ = 0.0815). The percentage of left- and right-hemispheric decisions neither correlated with the number of training trials nor with the percentage of correct responses at the end of training (Pearson correlations, ns).

Under monocular seeing conditions, most pigeons also showed a dominance of one hemisphere (Fig. 3A, B). However, contrary to our expectations, the pigeons did not choose more frequently those conflict stimuli that included positive colours, which were learned with the seeing eye. This should have resulted in a positive decision asymmetry when seeing with the right eye and a negative one when seeing with the left eye. However, this was not the case, neither on average nor for individual pigeons (Fig. 3A, B). A repeated measure ANOVA revealed that the percentage of hemispheric-specific decisions depended on the seeing conditions (F_2, 36_ = 9.823, *p* < 0.000; partial eta-squared η_p_^2^ = 0.353). Posthoc Bonferroni tests show that the percentage of left-hemispheric choices was significantly higher than right-hemispheric ones when seeing with both or with the right eye, but there was no difference in the percentage of hemispheric-specific decisions when seeing with the left eye (Fig. 4B). The percentage of right-hemispheric decisions did not differ at all between the viewing conditions (Fig. 4B).

**Fig. 5:**
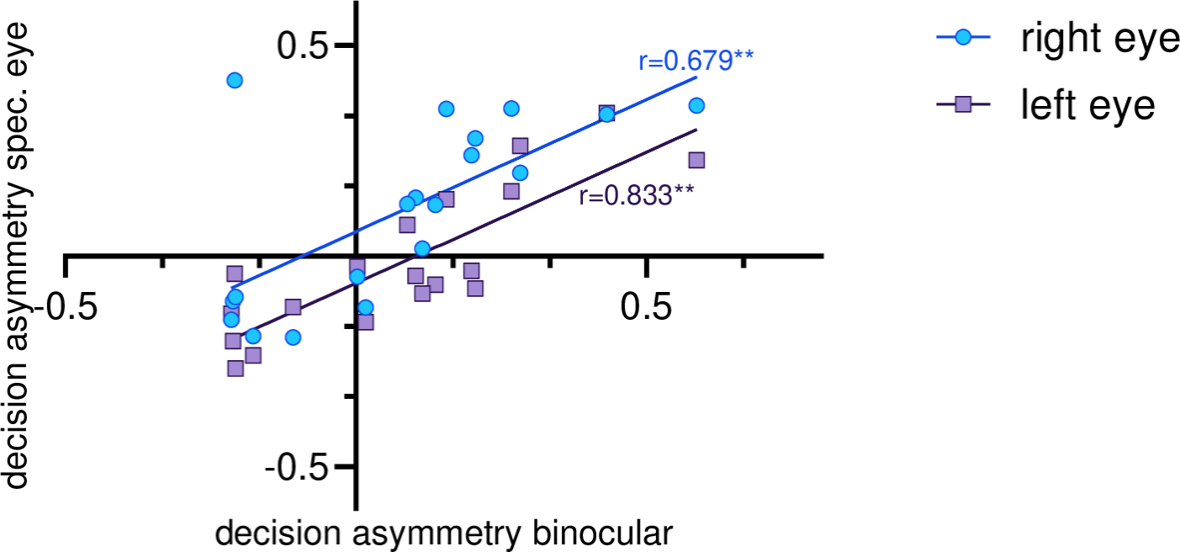
Correlations of decision asymmetry between binocular and monocular (left or right eye) seeing conditions. Note that the decision asymmetry correlates positively for all viewing conditions not negatively, indicating that the direction and not the strength of the decision dominances are associated (** = 0.001 according to Pearson correlation).

When comparing the decision asymmetries of individual animals under the different seeing conditions, it was striking that these asymmetries went in the same direction, regardless of the seeing conditions (Fig. 3A, B). Actually, asymmetry of conflict decisions correlated positively between the seeing conditions (Fig. 5). Binocular decision asymmetry correlated positively with the right-eye seeing (Pearson: r = 0.679, *p* = 0.001) and the left-eye seeing ones (Pearson: r = 0.833, *p* < 0.001); monocular decisions asymmetries also correlated positively (Pearson: r = 0.742, *p* < 0.001).

Individual metacontrol was not as frequent within the first binocular test session compared to all sessions though the absolute value of the mean decision asymmetry differed significantly from zero (one sample t-test: t = 4.957, *p* < 0.001; Fig. 3C, D). Only 30% (five light-exposed, one light-deprived) of the pigeons displayed metacontrol within the first session as indicated by significant differences between left- and right-hemispheric choices (Fig. 3C, D). Changes in response pattern were accompanied by an increase in the percentage of no-responses and a decrease in the mean percentage of conflict decisions (Fig. 4C, Supplementary Material 2 Fig. 1).

##### Reaction times

The analysis of binocular reaction times revealed no differences in mean response latencies between left- and right-hemispheric decisions, either within or between the groups with and without light exposure. In contrast to super stimuli discrimination, reaction times for conflict choices increased significantly over time (Supplementary Material 2 Fig. 2).

**Fig. 6:**
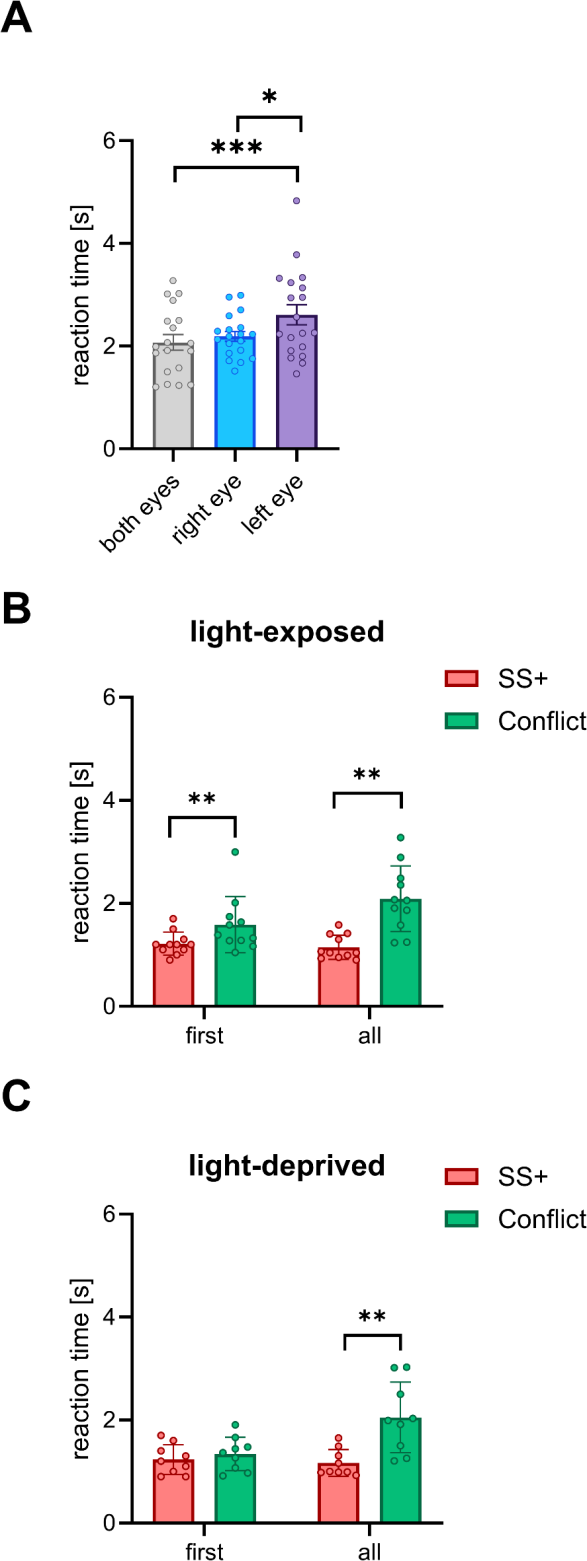
Mean reaction times under the different seeing conditions (**A**); comparison of response latencies for pecking onto correct superstimuli (SS +) and conflict stimuli during the first and all session in light exposed (**B**) and light-deprived pigeons (**C**) Bars represent standard error (* < 0.05, ** < 0.01, *** < 0.001 according to Wilcoxon tests).

A Friedman ANOVA evinced significant differences in reaction times between the viewing conditions (Chi^2^ (n = 19, df = 2) = 16.421 *p* < 0.0001). Under monocular viewing conditions, reaction times were faster when seeing with the right than with the left eye (Wilcoxon: z = − 2.294, *p* = 0.022; Fig. 6A). Reaction times with right eye vision did not differ from binocular ones (Wilcoxon: z = − 0.885, *p* = 0.376) in contrast to those with left eye vision (Wilcoxon: z = − 3.622; *p* < 0.001).

The analysis of response latencies can provide hints for the neuronal mechanisms mediating conflict decisions^25,26^. In this regard, it is intriguing that on average, reaction times were significantly slower for conflict decisions compared to decisions for SS + stimuli in both groups (Wilcoxon: z = − 3.920, *p* < 0.001; Fig. 6B, C). This was also the case for monocular conflict decisions (Wilcoxon: z = − 3.823, *p* < 0.001). However, this was different in the first binocular session. While in light-exposed birds, mean response latencies for conflict choices were slower than responses to SS + (Wilcoxon: z = − 2.845, *p* = 0.004; Fig. 6B), this was not the case for the light-deprived group (Wilcoxon: z = − 1.599, *p* = 0.110; Fig. 6C).

**Fig. 7:**
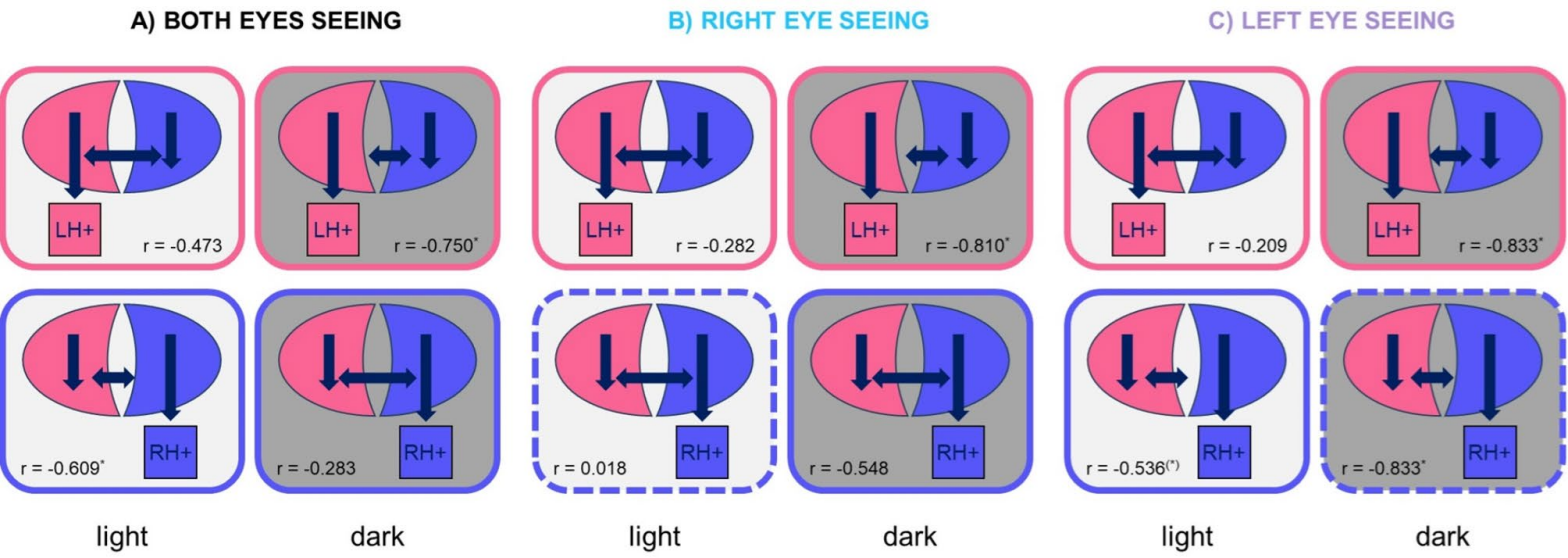
Potential role of intra- and interhemispheric mechanisms for hemispheric-specific decisions as indicated by correlations between the percentage of left- (LH + , pink frame) and right (RH + , blue frame)-hemispheric decisions and reaction times under the different seeing conditions in light-exposed (light grey boxes) and light-deprived (dark grey boxes) pigeons—indicated are correlation coefficients (r) and significance according to Spearman-Rho. Negative correlations indicate intrahemispheric mechanisms for controlling a response (short arrow). Absence of correlation indicate a critical role for interhemispheric mechanisms in controlling a response (long arrow). Left-hemispheric decisions are all controlled by interhemispheric mechanisms in light-exposed pigeons under all seeing conditions and by intrahemispheric mechanisms in light-deprived pigeons. Associations are reversed for right-hemispheric choices. There are only two exceptions (dashed frames)*.*

Moreover, correlation analyses indicated differential associations between the reaction times and hemispheric-specific responses in the two experimental groups. In light-exposed pigeons, the percentage of right-hemispheric decisions correlated negatively with response latencies (Spearman-Rho: r = − 0.609, *p* = 0.047, Fig. 7A) while there was no correlation for left-hemispheric choices (Spearman-Rho: r = − 0. 473, *p* = 0.142; Fig. 7A). This means that the percentage of right-hemispheric choices increased with faster reaction times while the percentage of left-hemispheric choices was independent from response latencies. For light-deprived pigeons, correlations displayed an opposite pattern with a negative correlation between left-hemispheric choices and reactions times (Spearman-Rho: r = − 0.750, *p* = 0.020, Fig. 7A) and no correlation between right-hemispheric decisions and reactions times (Spearman-Rho: r = − 0.283, *p* = 0.460, Fig. 7A). Comparable associations between hemispheric-specific choices and reaction times were also observed under monocular seeing conditions (Fig. 7B, C).

## Discussion

When confronted with interhemispheric conflict decisions, pigeons show metacontrol independent of embryonic light exposure, although hemispheric dominance is not necessarily evident in the first decision. Reaction time patterns indicate that interhemispheric mechanisms are basically involved in controlling metacontrol. However, individual left- and right-hemispheric decisions are probably differentially mediated by intra- and interhemispheric mechanisms, with their respective roles being exactly opposite in light-exposed and light-deprived pigeons. The underlying mechanisms even influence decision-making when seeing with only one eye and lead to hemispheric dominance independent of the viewing conditions. This indicates a remarkable interhemispheric communication between the hemispheres when confronted with conflict decisions.

### Stimulus discrimination during training and conflict phase

In order to understand whether metacontrol is based on mechanisms specific to conflict decisions, it is first necessary to check whether the left- and right-eye systems differ in their learning performances and discrimination accuracies. Since some (albeit not all) previous studies report asymmetrical learning of colour discriminations in pigeons^24,28,38^, it is possible that advantages in visual processing or learning cause the dominance of one hemisphere for conflict choices. In our study, however, we did not observe any left–right differences in learning or discrimination of the trained colour stimuli. Comparable learning performances of the two eye systems were confirmed by the absence of left–right differences in the discrimination of the superstimuli during the monocular conflict tests. Furthermore, neither the number of training trials nor the discrimination accuracy with the left or right eye correlated with the percentage of left- and right-hemispheric conflict decisions. All this makes it highly unlikely that associative mechanisms alone account for the dominance of one hemisphere and supports that metacontrol emerges by neuronal mechanisms specific for conflict decisions^25^(see below).

### Metacontrol pattern in light-exposed and -deprived pigeons

In our study, a majority of pigeons displayed individual metacontrol so that not the mean but the absolute value of the decision asymmetry was significant. There are already some publications describing hemispheric dominance in conflict choices based on colour discrimination. However, only one study reported a left-hemispheric dominance at the group level^24^, while all other studies showed individual metacontrol, but only for a minority of the birds tested^23,26,27^. These discrepancies lead to the question of whether there are test conditions or factors which influence the emergence of hemispheric dominance in conflict situations.

One factor could be the developmental light experience since in birds, ontogenetic light exposure affects hemispheric asymmetries as well as interhemispheric communication^31,32^. It is therefore remarkable that, in contrast to our original assumption, not only light-exposed but also light-deprived pigeons displayed hemispheric dominance for conflict decisions. Thus, metacontrol is an additional example for brain asymmetries, which is not dependent on asymmetrical ontogenetic light experiences^11,13,14,36^. This suggests that hemispheric dominance for conflict decisions is essentially an inherent characteristic of the pigeon`s brain. However, this does not explain why not all studies report metacontrol in a majority of the tested pigeons. We suggest that differences in the experimental procedure could provide a plausible explanation.

The higher frequency of metacontrol in our experiment might be caused by increased statistical validity since our results are based on a much higher number of conflict decisions in a higher number of sessions compared to previous studies. In this regard, it is intriguing that in the first session, in which the pigeons already conducted as many pecking responses as in previous studies, only a smaller proportion of birds (about 30%) showed metacontrol. This percentage is fully in line with the other studies^23,26,27^. However, a closer look onto the conflict decision pattern over time indicates that not simply the higher amount of pecking decisions accounted for increased individual metacontrol. Since the percentage of non-responding increased, the mean percentage of conflict decisions decreased. This was presumably the result of extinguishing responses towards the non-rewarded conflict choices. Although we designed the conflict session in such a way that differences between super and conflict stimuli were less obvious, all pigeons decreased pecking specifically onto conflict but not onto super stimuli over time. Thereby, specifically the percentage of right- but not left-hemispheric decisions decreased what in turn affected the balance of left- and right-hemispheric decisions and therefore, hemispheric dominance. This indicates that metacontrol patterns are not static and hemispheric dominance becomes only stable after a sufficient number of pecking responses.

### Hemispheric dominance and seeing conditions

For birds with laterally placed eyes, several hints from behavioural and electrophysiological studies indicate that the hemispheres process incoming visual input primarily independent from each other^16,39,40^ with only delayed interhemispheric transfer^41^. Thus, metacontrol should appear specifically under binocular seeing conditions when both hemispheres receive visual input in parallel and therefore directly compete for decision-making and response control. When seeing with only one eye, the hemisphere contralateral to the seeing eye has direct access to the visual input and hence, a processing advantage, which should enable this hemisphere to control the pecking response. Consequently, we expected the pigeons to choose more often the stimuli including the positive colours of the seeing eye and it was very surprising that the pigeons did not show such a decision bias. However, there were differences when seeing with the left or right eye, which were similar for pigeons with and without embryonic light experience. When seeing with the right eye, there was at least a trend for the expected higher proportion of left-hemispheric decisions. This confirmed a processing advantage of the left hemisphere under monocular seeing conditions^28–30,38^. In contrast, there was absolutely no difference in the percentage of left- and right-hemispheric choices when seeing with the left eye and there was also no difference in the percentage of right-hemispheric decisions between the two seeing conditions. Thus, the right hemisphere does not seem to benefit from a more direct access to visual input. This difference is also supported by the slower reaction times of left-eye choices compared to binocular and right-eye ones.

Nevertheless, for the majority of pigeons it could be the left or the right hemisphere, which dominated conflict decisions whereby monocular and binocular decision asymmetries correlated positively (rather than negatively). Thus, it was the direction and not the strength of hemispheric dominance, which was consistent between viewing conditions. All of this suggests that the hemisphere, which exerts metacontrol when seeing with both eyes also tends to control the monocular decisions, even when it does not have direct access to the ambiguous information. This strongly suggests that interhemispheric communication in the pigeon brain is much more pronounced than generally thought^42–44^.

### Neuronal mechanisms underlying metacontrol

Theoretical models propose a differential role of intra- and interhemispheric mechanisms for the generation of metacontrol, which can be indirectly supported by the analysis of reaction times. On the one hand, left–right differences in intrahemispheric processing speed can lead to metacontrol if one hemisphere gains faster control over network mediating decision-making and/or motor control. If such a processing advantage is independent of the task conditions, reaction times for conflicting decisions should not differ from those for non-conflicting ones. On the other hand, asymmetries of interhemispheric interactions can be critical for the emergence of metacontrol, with one hemisphere gaining dominance by influencing processing on the contralateral side more efficiently than vice versa. This additional interhemispheric processing step should prolong reaction times specifically for conflict decisions^18,25,26^.

As already discussed in the previous section, it is likely that interhemispheric components control conflict decision-making what enables the dominant hemisphere to exert metacontrol even under monocular viewing. The critical role of interhemispheric mechanisms is supported by the longer response latencies for conflict decisions compared to the discrimination of the superstimuli. The impact of interhemispheric mechanisms may not be surprising given that the response selection in our task was not simply a decision for one of the two conflict stimuli (the one that including a positive colour and hence, had an appetitive value for the dominant hemisphere), but explicitly against the decision of the minor hemisphere, for which the same stimulus had an aversive value because it included a negative colour. However, changes in response pattern and latencies indicate a more complex interplay of intra- and interhemispheric mechanisms, whose dynamics differ between light-exposed and light-deprived pigeons. Light-exposed pigeons displayed slower reaction times for conflict decisions already in the first session. This supports the role of interhemispheric mechanisms for decision-making from the first choices onwards. In light-deprived pigeons on the contrary, reaction times for conflict decisions did not differ from those for superstimulus discrimination during the first session suggesting that intrahemispheric mechanisms govern initial pecking decisions in this group. As almost no pigeon (except of one) of the light-deprived group showed metacontrol during the first conflict session, none of the hemispheres seem to have a general processing advantage and intrahemispheric mechanisms alone are not sufficient for the generation of a consistent hemispheric dominance. Since prolonged reaction times only emerge during additional test sessions, interhemispheric mechanisms presumably only play a role with increasing experience.

Despite a general dominance of one hemisphere, all pigeons show left- as well as right-hemispheric choices during one session. Although we do not know what accounted for a decision in a single trial, it is likely that left- and right-hemispheric choices are based on different decision strategies, which are mediated by different computational processes. There are hints that the right hemisphere ignores information from the left hemisphere in case of interhemispheric conflict and therefore, response control should be based on intrahemispheric mechanisms. The left hemisphere on the other hand considers and compares input from both brain sides^45^ and thus, especially left-hemispheric dominance should include interhemispheric components^25,26^. Correlations between hemisphere-specific decisions and reaction times actually support a different influence of intra- and interhemispheric processes for left- and right-hemispheric decisions. According to an influential model explaining visual asymmetries in birds by Xiao & Güntürkün^28–30^, the left hemisphere of the pigeon brain has a principal processing advantage because of a neuronal organisation, which induces higher activation of intrahemispheric visual circuits^24,28,38^. This alone may enable the left hemisphere to dominate visuomotor processing^24^. In addition, the left hemisphere increases its processing advantage by slowing down neuronal processing in the right forebrain via commissural fibres. As a result, the neurons of the right hemisphere come too late to control a response and the left hemisphere governs a decision. Conversely, the right hemisphere cannot influence left-hemispheric processing^28^. This means that the left hemisphere can achieve decision dominance via both intra- as well as interhemispheric processes, which enables it to take over response selection independent from processing speed. In contrast, right hemispheric dominance must result solely from intrahemispheric mechanisms. This means that the right hemisphere can only dominate a conflict decision if it generates a decision response faster than left-hemispheric influences can intervene^25^. These differences become evident when correlating hemispheric-specific decisions with reaction times^25^. In our study, there was no correlation between left-hemispheric choices and reaction times in light-exposed pigeons, which is fully in line with the model of Xiao & Güntürkün^28^. In contrast, there was a negative correlation between reaction times and right-hemispheric choices. This means that the faster a pecking response was conducted, the higher was the proportion of right-hemispheric decisions. This association is therefore also consistent with the model of Xiao & Güntürkün^28^. Exactly the same correlations are reported for metacontrol patterns based on categorization^25^. Intriguingly, interrelations between hemispheric-specific choices and reactions times were reversed in light-deprived pigeons. There was no correlation between right-hemispheric choices and reaction times but a negative one between left-hemispheric choices and reaction times. Thus, in light-deprived pigeons, it is the left hemisphere that must be fast to control a conflict decision indicating that the left hemisphere has no efficient access to interhemispheric mechanisms. This in turn implies that left-hemispheric dominance of interhemispheric control depends on embryonic visual stimulation, which is consistent with previous reports showing that ontogenetic light exposure alters the direction and efficiency of interhemispheric mechanisms^11–15,36^. In pigeons, embryonic light stimulation increases left-hemispheric access to interhemispheric visual information, reversing the light-independent dominance of the right hemisphere^13^. Since the left-hemispheric dominance develops in parallel with increased bilateral visual input^9^, it is conceivable that a resulting stronger left-hemispheric neuronal activation enables increased control over interhemispheric decisions. In light-deprived pigeons on the other hand, there are no left–right differences in visual input^9^ and thus, no asymmetry of neuronal activation emerges, which could modify the endogenous right-hemispheric superiority in using interhemispheric mechanisms. The strength of the influence of lateralized interhemispheric mechanisms is supported by the fact that almost identical correlations were observed in both experimental groups under monocular viewing conditions (Fig. 7).

In sum, ontogenetic light experiences are not necessary for the emergence of metacontrol itself but seem to influence the dynamics of the underlying processes. Asymmetric light stimulation leads to structural left–right differences within the ascending visual system, such that the left hemisphere is more strongly activated by visual input in light-exposed pigeons, while the two hemispheres of light-deprived pigeons are activated more symmetrically^9^. This difference is not necessarily responsible for the dominance of one hemisphere (in fact, there were more light-deprived pigeons showing left-hemispheric metacontrol), but rather affects the relative influence of the underlying lateralised intra- and interhemispheric mechanism.

## Conclusions

In summary, our study provides important evidence for the functional organisation of metacontrol in birds. A majority of pigeons displayed hemispheric dominance for conflict choices, verifying that metacontrol is a relevant decision-making process in animals with laterally placed eyes. In most pigeons, however, neither hemisphere dominated the decisions already during the first conflict trials. Only with increasing experimental experience did one hemisphere gain dominance. The emergence of metacontrol is accompanied by fewer and slower responses. This suggests that metacontrol is not primarily a mechanism enabling fast reactions in new ambiguous situations. It is therefore plausible that metacontrol is caused by a more elaborated experience-based evaluation of the potential choices^24^ and is related to extinction learning of uncertain decisions^46^.

Interhemispheric mechanisms seem to be involved here, which are also crucial under monocular visual conditions for one hemisphere to control response selection, even when it receives no direct visual input. Metacontrol thus shows that interhemispheric communication in the avian brain is a highly dynamic process, which influences decision-making more strongly than previously assumed. Associations between decision pattern and reaction times indicate that left- and right hemispheric choices are based on divergent processing strategies, which differentially depend on interhemispheric mechanisms. Differences between light-exposed and light-deprived pigeons indicate that embryonic light exposure influences these dynamics and provide additional evidence for the critical role of asymmetrical sensory experience in the emergence of a lateralised functional organisation of the brain.

## Material & methods

### Subjects

We used eleven adult pigeons (*Columba livia*) from local breeders or from lab-owned breeding pairs as well as nine adult dark-incubated animals from lab-owned breeding pairs for this study (Table 1). Sex of the animals was not determined. For dark-incubation, fertilized eggs from pairs of breeding pigeons were incubated in still-air incubators kept in darkness at a constant temperature (38.3 °C) and humidity (60–75%) throughout the entire period of incubation. Directly after hatching, the nestlings were banded and swapped with the artificial eggs the breeding birds were sitting on^9,37^.

**Table 1:** Experimental animals.

	Experimental group	Right-eye system		Left-eye-system		Binocular conflict tests	Monocular conflict tests
		S +	S −	S +	S−		
T400	Light-exposed	Set 2	Set 1	Set 4	Set 3	16	12/12
T628	Light-exposed	Set 3	Set 1	Set 2	Set 4	16	12/12
T265	Light-exposed	Set 1	Set 2	Set 3	Set 4	16	12/12
682T	Light-exposed	Set 3	Set 4	Set 1	Set 2	16	12/12
T155	Light-exposed	Set 1	Set 4	Set 2	Set 3	16	12/12
T603	Light-exposed	Set 2	Set 3	Set 4	Set 1	16	12/12
T470	Light-exposed	Set 1	Set 2	Set 3	Set 4	16	12/12
T838	Light-exposed	Set 4	Set 3	Set 1	Set 2	16	12/12
T839	Light-exposed	Set 4	Set 1	Set 2	Set 3	16	12/12
T069	Light-exposed	Set 2	Set 4	Set 1	Set 3	16	12/12
T999	Light-exposed	Set 4	Set 1	Set 3	Set 2	16	12/12
T667	Light-deprived	Set 2	Set 1	Set 4	Set 3	16	12/12
T730	Light-deprived	Set 4	Set 2	set 1	set 3	12	0/0
T731	Light-deprived	Set 1	Set3	Set 2	Set 4	16	12/12
T732	Light-deprived	Set 4	Set 3	Set 2	Set 1	16	12/12
T734	Light-deprived	Set 3	Set 1	Set 4	Set 2	16	12/12
T735	Light-deprived	Set 3	Set 1	Set 4	Set 2	16	12/12
T736	Light-deprived	Set 2	Set 4	Set 3	Set 1	16	12/12
T737	Light-deprived	Set3	Set 4	Set 1	Set 2	16	12/12
T738	Light-deprived	Set 1	Set 2	Set 3	Set4	16	12/12

The birds were maintained on a 12-h light–dark cycle. Water and grit were freely available while food was restricted to keep the weight at 85% of free-feeding weight. Food was provided daily during training and after the sessions when necessary. Each bird conducted one session per day (5 days per week) under different seeing conditions depending on the experimental phase. To restrict view during monocular sessions, rings of Velcro (soft part) were fixed around the pigeons’ eyes using non-toxic glue (UHU Crafts glue). In this way, cardboard caps with the Velcro hook counterpart glued on the inside could be easily attached and removed^25,45^.

The experiments were carried out in compliance with the European Communities Council Directive of September, 22 2010 (2010/63/EU) and the specifications of the German law for the prevention of cruelty to animals and was approved by the animal ethics committee of the Landesamt für Natur, Umwelt und Verbraucherschutz (LANUV) NRW, Germany. We confirm that all methods were carried out in accordance with relevant guidelines and regulations and that the study was conducted in compliance with the ARRIVE guidelines.

### Apparatus and materials

Pigeons were trained and tested in a custom-made operant chamber (35 × 39 × 39 cm^3^). Two LED strips illuminate the chamber, which were turned off as mild punishment during training. Stimuli with the size of 5 × 5 cm were presented on a TFT LCD touchscreen monitor (mode l ET1515L with APR technology, Elo Touch Solutions, Inc., Milpitas, CA, USA, with 1024 × 768 resolution) fixed to the back of the chamber. Centrally below the touch screen, a food hopper delivered mixed grain as reward. Experimental sessions were controlled and recorded by custom-written MATLAB programs (MathWorks, Natick, MA, USA) using the Biopsy Toolbox^47,48^.

### Stimuli

For the colour discrimination training, four different sets of stimuli were created whereby each set included two colours. The stimuli consisted of a white and a coloured half, or combined two colours of one stimulus set (Fig. 1). Therefore, the pigeons were familiarized already during the training phase with “double-coloured” stimuli, which were presented during conflict sessions. The colours of the sets had two different RGB grey gradations, the intensity of which did not differ between the different colour sets. Each eye was trained with different sets whereby positive (rewarded) and negative (unrewarded) colour sets were randomized between pigeons and eye systems (Table 1). For the conflict tests, the colours were combined, so that all stimuli consisted of two colours each filling one half of the stimulus. Two different types of stimuli were presented during the conflict trials. First, two colours of the positive sets for the left- and right-eye systems were combined to create positive superstimuli (SS +), which had to be discriminated from negative (SS−) stimuli, which combined two negative colours from the left- and right-hemispheric sets (Fig. 1B)^26^. Second, a positive colour for one and a negative one for the other eye system were combined to generate ambiguous stimuli (Fig. 1C). Two of these ambiguous stimuli were randomly paired during conflict choice sessions. In all tests, each stimulus could appear in two versions with reversed top–bottom colour orientation.

### Training and testing procedure

First, the pigeons learned in an autoshaping procedure that pecking on a white stimulus resulted in food supply^24–26^. Second, animals were trained in colour discriminations until reaching the learning criterion in a forced choice task. Third, the pigeons completed conflict tests under binocular and fourth, monocular seeing-conditions (Fig. 1D). The pigeons were trained 5 days a week, with the eye conditions alternating between consecutive days.

For colour discrimination training, pigeons had to discriminate the described above stimuli under monocular seeing conditions (Fig. 1). Training was divided into three phases. In phase 1, the pigeons were confronted with the first colour pair (P1, one positive, one negative). After reaching the learning criterion of 85% correct responses two times in a row under both seeing conditions, phase 2 started by introducing the second positive and negative colours (P2). Trials of a training session included 50% P1 and 50% P2 stimuli. After reaching the learning criterion, phase 3 started by introducing the double-coloured (P3) stimuli. Trials of a session included 30% P1, 30% P2 and 40% P3 stimuli. After reaching the learning criterion, reward rate was reduced gradually from 100 to 40%. In parallel, percentage of presented stimulus types was changed to 10% P1, 10% P2 and 80% P3 stimuli since only double-coloured stimuli appeared during conflict sessions. When the pigeons reached the learning criterion under a 40% reward rate, the conflict test phase began. This last P3 training procedure was also used during the training sessions, which were interleaved between the different conflict sessions during the conflict test phase.

Each training session consisted of 150 trials whereby up-down position of positive and negative stimuli was balanced over the trials of a session. A trial was indicated by a short tone and started with the presentation of a white initialization key, which had to be pecked once within a 4 s time window. After a 2 s inter-trial interval (ITI), a stimulus pair combining a positive (S +) and a negative (S −) stimulus appeared for a fixed time interval of 8 s. When a correct choice was made (single peck on S +), the display disappeared and eventually (in 40–100% of the trials depending on the experimental phase) reward was given (2–4 s access to food and feeding light on); incorrect choices (single peck on S −) resulted in a time out of 4 s with the house light turned off. An 8 s ITI followed each trial. Each peck during ITI prolonged ITI additional 2 s.

A conflict session consisted of 200 trials whereby 120 trials showed super stimuli and 80 trials conflict stimulus pairs. Thereby, super stimuli responses were partly and none of the conflict choices were rewarded to achieve a reward rate of 40%. Just like the discrimination training, a trial started with a tone and the appearance of a white initialization key. After an ITI of 2 s, either super stimulus or conflict stimulus pairs were presented for a fixed interval of 10 s. Responses were eventually rewarded and the next trial started after an ITI of 8 s.

Six binocular conflict test sessions alternated with monocular training sessions until a pigeons finished 16 test sessions with an initialization rate higher than 75%. After an additional training phase, monocular conflict test phase started with alternating eye conditions each day (4–6 sessions in a row) alternated with monocular training sessions until a pigeons finished 12 test session with an initialization rate higher than 75% for each eye (Fig. 1D).

### Analysis

Statistical analysis was conducted with IBM SPSS Statistics 21. We analysed percent pecking decisions (in relation to initialization rate) and reaction times within single sessions. Decision asymmetry was calculated as (left-hemispheric decision–right-hemispheric decision)/(left-hemispheric decision + right-hemispheric decision). To analyse hemispheric dominance specifically within the first session, we grouped conflict decisions into blocks of ten responses and compared left- and right-hemispheric decisions and reaction times.

Normal distribution was evaluated by Kolmogorov–Smirnov and Shapiro–Wilk tests and homogeneity of variance by Levene tests. For normally distributed data, we conducted two-tailed parametric tests (pecking decisions), otherwise non-parametric tests (reaction times, pecking decisions within the first session). We always compared data of the light-exposed and light-deprived group and combined data sets for statistical analysis when there was no difference between the groups.

## Supplementary Information

Supplementary Information 1.

Supplementary Information 2.

## Data Availability

All individual data are included into the supplementary material. Original matlab files are currently available from the corresponding author upon request.
