# Publisher Correction: Dynamics and development of interhemispheric conflict solving in pigeons

**DOI:** 10.1038/s41598-025-92470-2

**Published:** 2025-04-04

**Authors:** Martina Manns, Kevin Haselhuhn, Nadja Freund

**Affiliations:** 1 Division of Experimental and Molecular Psychiatry, Department of Psychiatry, Psychotherapy and Preventive Medicine, LWL University Hospital, Ruhr-University, Bochum, Germany; 2 Department of Biopsychology, Faculty of Psychology, Institute of Cognitive Neuroscience, Ruhr-University Bochum, Bochum, Germany

Correction to: *Scientific Reports* 10.1038/s41598-024-85058-9, published online: 11 January 2025

In the original version of this Article, Figures 2, 4 and 6 were incorrectly displayed.

**Fig. 2:**
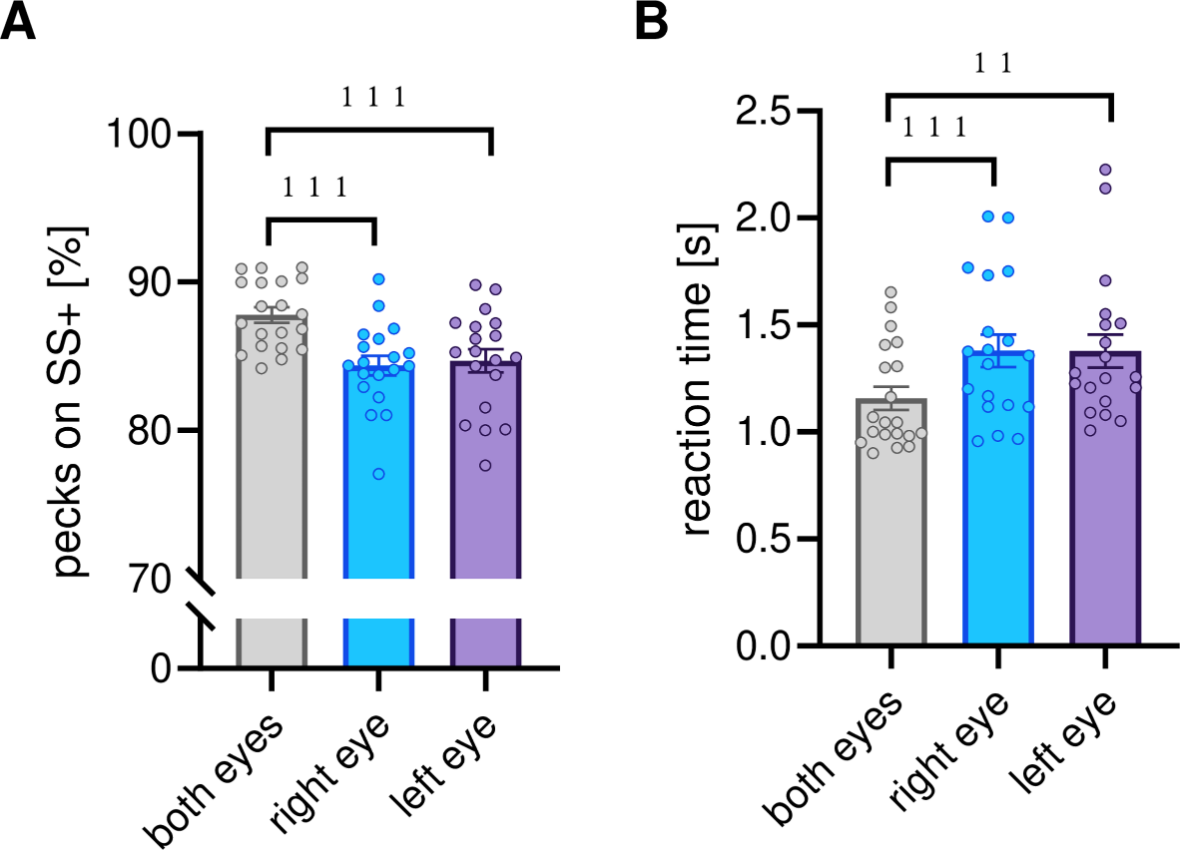
Super stimuli discrimination under different seeing conditions—(**A**) mean percent decisions for positive superstimuli (SS +); (**B**) mean reaction time in seconds [s] when pecking onto SS +. Bars indicate standard error, circles indicate individual data; ***p < 0.001, **p < 0.01 according to *t*-tests for dependent samples (**A**) or Wilcoxon tests (**B**).

**Fig. 4:**
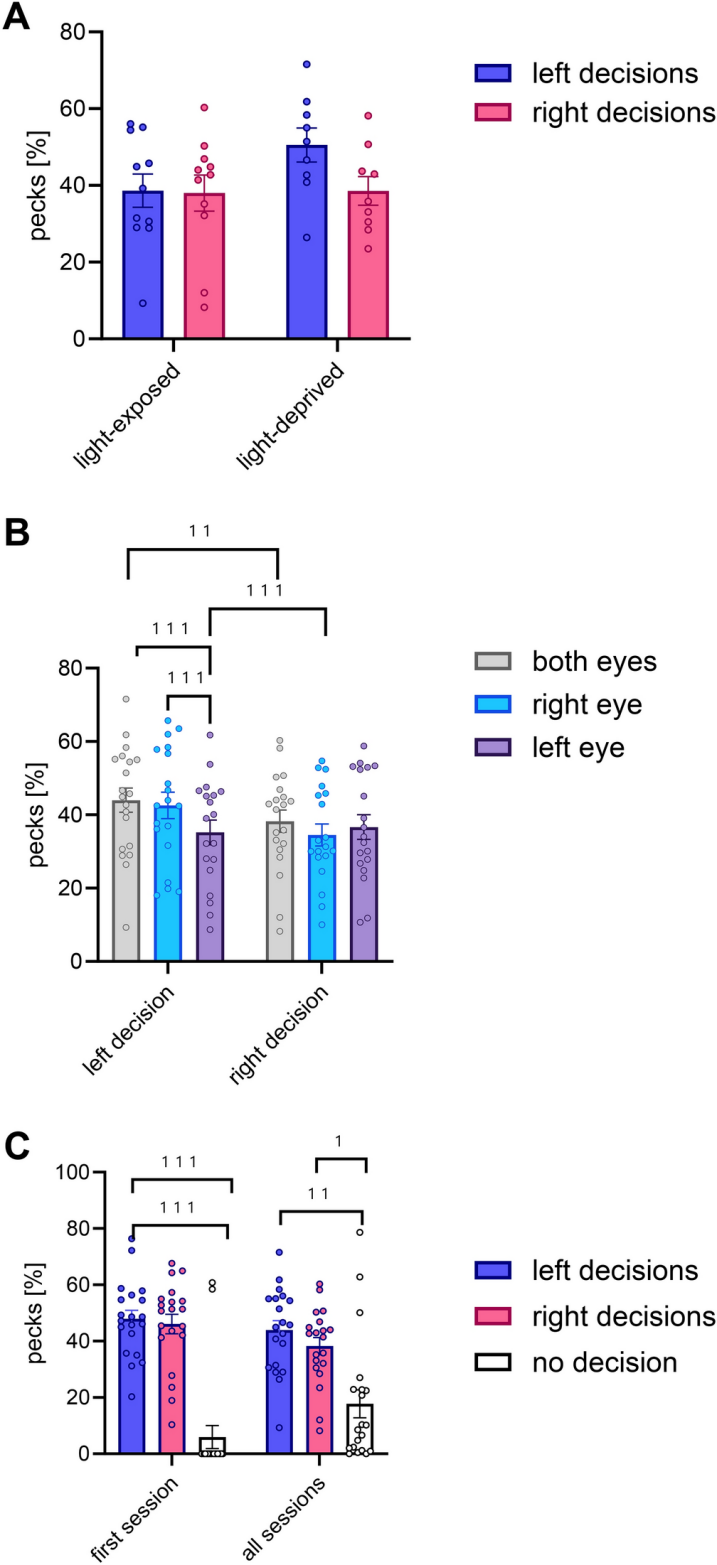
Hemispheric-specific decisions under binocular seeing conditions in light-exposed and light-deprived pigeons (**A**), under different seeing conditions (**B**), during first and all session (**C**). Bars represent standard error; (*) < 0.1, * < 0.05; **p 0 < 0.01, *** = *p* < 0.001 according to posthoc Bonferroni tests (**B**), Wilcoxon test (**C**).

**Fig. 6:**
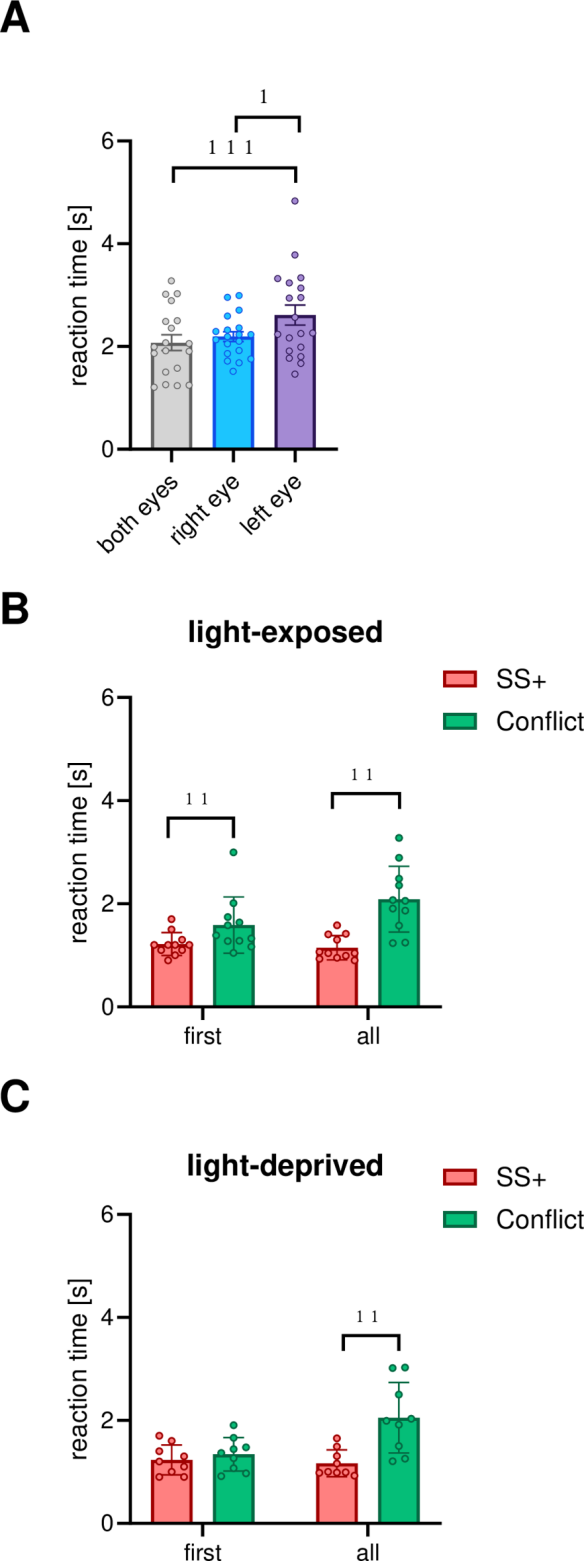
Mean reaction times under the different seeing conditions (**A**); comparison of response latencies for pecking onto correct superstimuli (SS +) and conflict stimuli during the first and all session in light exposed (**B**) and light-deprived pigeons (**C**) Bars represent standard error (* < 0.05, ** < 0.01, *** < 0.001 according to Wilcoxon tests).

The original Figures 2, 4 and 6 and the accompanying legends appear below.

The original Article has been corrected.

