# Supplementary material for: Dynamics and development of interhemispheric conflict solving in pigeons
Source: Sci Rep. 2025 Jan 11;15:1655. doi: 10.1038/s41598-024-85058-9 (PMC11723973; doi:10.1038/s41598-024-85058-9)
Supplement: Supplementary file 2 — Supplementary Information 2. [file 41598_2024_85058_MOESM2_ESM.pdf]

Supplementary Material 2

Dynamics and development of interhemispheric conflict solving in pigeons

Martina Manns[1], Kevin Haselhuhn[2], Nadja Freund[1]

[1]Department of Psychiatry, Psychotherapy and Preventive Medicine, LWL University Hospital, Ruhr-University, Bochum, Germany

[2] Department of Biopsychology, Institute of Cognitive Neuroscience, Faculty of Psychology, Ruhr-University Bochum, Bochum, Germany

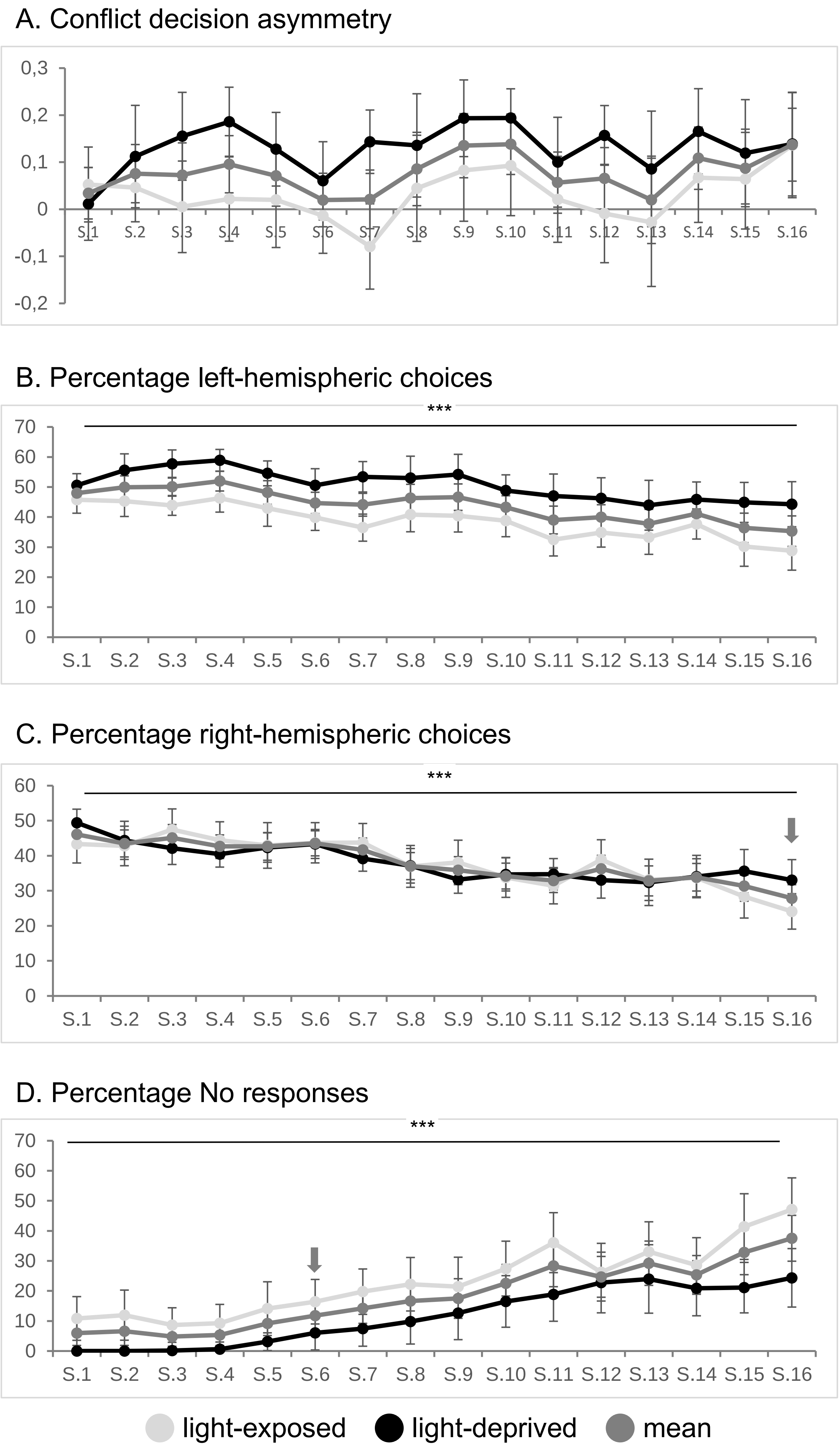
A. Conflict decision asymmetry
0,3
0,2
0,1
0
-0,1
-0,2
S.1 S.2 S.3 S.4 S.5 S.6 S.7 S.8 S.9 S.10 S.11 S.12 S.13 S.14 S.15 S.16
B. Percentage left-hemispheric choices
***
70
60
50
40
30
20
10
0
S.1 S.2 S.3 S.4 S.5 S.6 S.7 S.8 S.9 S.10 S.11 S.12 S.13 S.14 S.15 S.16
C. Percentage right-hemispheric choices
***
60
50
40
30
20
10
0
S.1 S.2 S.3 S.4 S.5 S.6 S.7 S.8 S.9 S.10 S.11 S.12 S.13 S.14 S.15 S.16
D. Percentage No responses
***
70
60
50
40
30
20
10
0
S.1 S.2 S.3 S.4 S.5 S.6 S.7 S.8 S.9 S.10 S.11 S.12 S.13 S.14 S.15 S.16
light-exposed
light-deprived
mean

Figure S1: Conflict decision pattern over all 16 binocular sessions as indicated by decision asymmetry (A.), percentage of left-hemispheric (LH; B.), right-hemispheric (RH, C.) choices and percentage of No responses (D.). Depicted are the mean data of all pigeons as well as those of light-exposed and light-deprived pigeons separately. A repeated measure ANOVA (including data of all pigeons) with “session” as repeated measure factor indicates no change over time for choice asymmetry (A. F = (1, 15) = 0734, p= 0.635 (Greenhouse-Geisser corrected), partial eta-squared (η_p^2) = 0.041) but for the percentage of left- (B. F = (1, 15) = 4.545, p= 0.001 (Greenhouse-Geisser corrected), η_p^2= 0.202) and right- (C. F = (1, 15) = 5.267, p< 0.001 (Greenhouse-Geisser corrected), η_p^2=0.226) hemispheric choices. Bonferroni corrected posthoc tests show a significant difference between the first and 16th session for right hemispheric choices (arrow in B.). For the percentage of No responses (D.), a Friedman ANOVA also indicates a session effect (Chi^2 (n=19, df=15) = 127.023, p < 0.001). Posthoc Wilcoxon test indicates a significant difference of the first to all other session from the 6th session onwards (arrow in D). Bars indicate standard error.

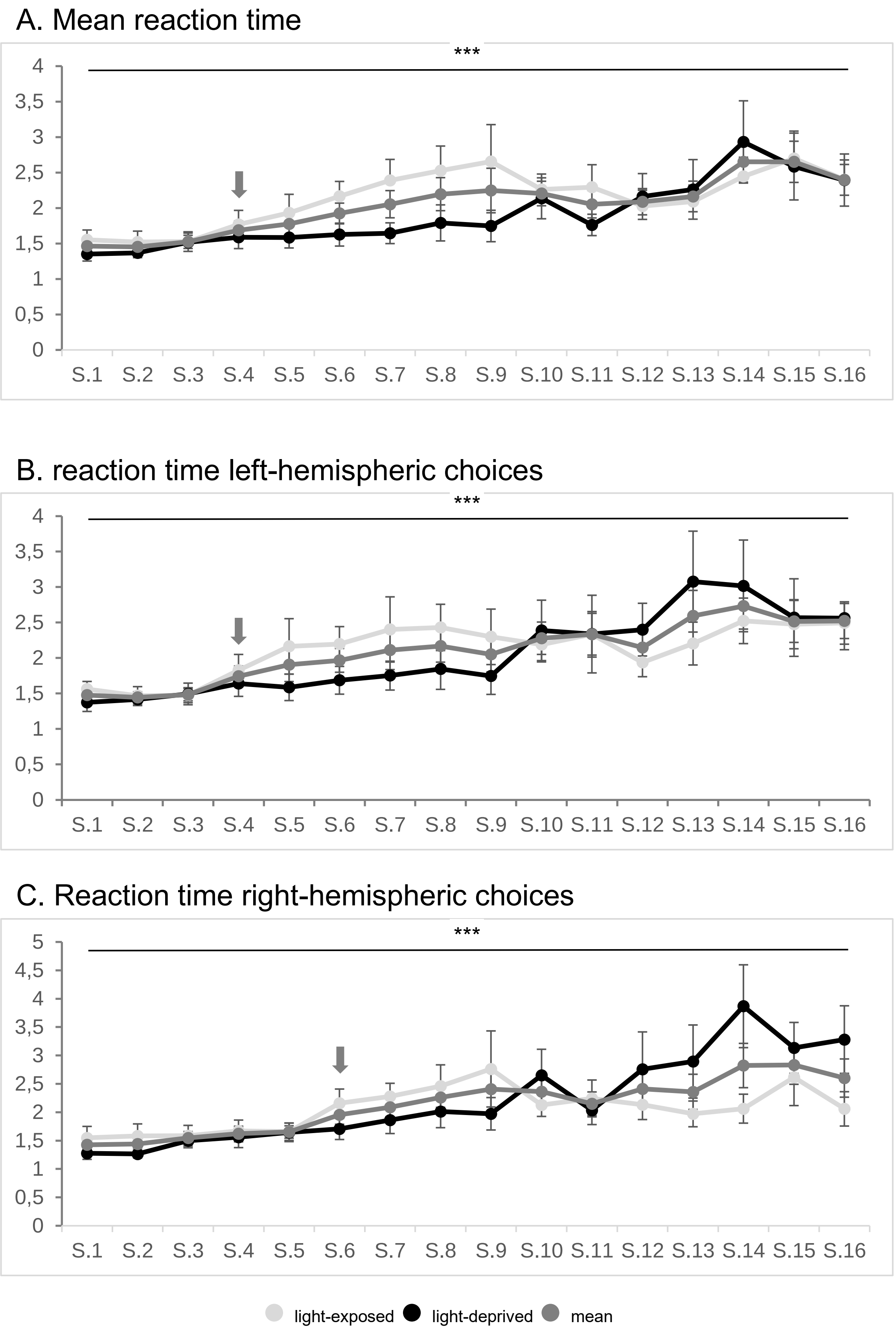

Figure S2: Reaction times over all 16 binocular sessions (A.) as well as reaction time specifically for left- (B.) and right- (C.) hemispheric choices: Depicted are the mean data of all

as well as those of only light-exposed or light-deprived pigeons. Friedman ANOVAs (including data of all pigeons) indicate a session effect for reaction times (Chi^2 (n=19, df=15) = 127.023, $p < 0.001$ in A., B., C.). Posthoc Wilcoxon tests indicate a significant difference of the first session (arrows) to all other session from the 4th (A., B.) or 6th (C.) session onwards. Bars indicate standard error.
